# Rapid Diagnosis of Pandemic (H1N1) 2009 in Cuba

**DOI:** 10.3201/eid1802.110547

**Published:** 2012-02

**Authors:** Belsy Acosta, Alexander Piñón, Odalys Valdés, Clara Savón, Amely Arencibía, Elías Guilarte, Gonzalez Grehete, Suset Oropesa, Gonzalez Guelsys, Bárbara Hernández, Angel Goyenechea, Mayra Muné, Vivian Kouri, María G. Guzmán, Alina Llop

**Affiliations:** Instituto Pedro Kourí, Havana, Cuba

**Keywords:** pandemic (H1N1) 2009, influenza, viruses, surveillance, rapid diagnosis, Cuba

**To the Editor:** During 2005–2008, the Cuban National Influenza Center (NIC) at the Pedro Kourí Institute in Havana, Cuba, implemented a protocol for influenza surveillance proposed by the Pan American Health Organization and the US Centers for Disease Control and Prevention (*1*). One of the most essential features of this protocol was strengthening laboratory capacity for surveillance of seasonal influenza and timely detection of a new influenza virus with pandemic potential.

On April 26, 2009, in response to an outbreak of pandemic (H1N1) 2009 in Mexico, NIC proposed an algorithm with 2 phases for processing specimens. The purpose of the algorithm was to identify the novel virus, effectively monitor its circulation in Cuba, and make these data available to the national health authorities and the World Health Organization (WHO) (*2*).

The first phase of the algorithm was used during April–September 2009. Following the recommendations of WHO (*3*), this phase included fluorescent antigen tests, nucleic acid extraction by using QIAamp Viral RNA and QIAamp Viral DNA Kits (QIAGEN, Hilden, Germany), and 3 reverse transcription PCR (RT-PCR) assays for typing and subtyping of influenza A viruses. RT-PCRs were used for differential diagnoses, which included 15 other respiratory viruses (*4**–**7*).

At the same time, on the basis of pandemic (H1N1) 2009 virus sequences published on the Global Initiative on Sharing Avian Influenza Data website (http://platform.gisaid.org/dante-cms/struktur.jdante?aid=1131), we designed a primer set specific for the hemagglutinin gene and we developed an in-house, conventional RT-PCR was designed to enable virus-specific identification. Confirmation was performed by subsequent sequencing of hemagglutinion, nucleoprotein, or neuraminidase genes by using the BigDye Terminator Cycle Sequencing Quick Start Kit (Beckman Coulter Inc., Krefeld, Germany).

Several sequences obtained were submitted to GenBank under accession nos. HM159409–159418 and HM176606–HM17639) (*8**,**9*). BLAST (www.ncbi.nlm.nih.gov/BLAST/) search analysis on sequences obtained from the first cases identified indicated the highest alignment score (>98% identity) with pandemic (H1N1) 2009 virus strains.

The second phase of the algorithm was used during September 2009–August 2010. This phase included automated nucleic acid extraction (QIAcube; QIAGEN) and real-time RT-PCR kits (Pan American Health Organization, Washington, DC, USA, and Centers for Disease Control and Prevention, Atlanta, GA, USA) for detection and characterization of pandemic (H1N1) 2009 virus (*10*).

During January 3–July 31, 2009, a total of 2,156 specimens were submitted to the NIC for influenza surveillance. During January 2009–August 10, 2010, a total of 14,692 clinical samples were processed. In the next 6 months, the number of specimens submitted to NIC doubled. More specimens (7,978) were submitted during January 1–August 10, 2010, than during all of 2009.

Most (5,601) clinical specimens processed were from patients with influenza-like illness. The highest percentage (45.9%) of influenza-positive samples was detected in specimens from these patients, followed by specimens from patients during outbreaks (18.0%).

Pandemic (H1N1) 2009 virus infection peaked during epidemiologic weeks 39–41, 2009 (Figure A1) and co-circulated with influenza A virus (H3N2). This period coincided with the start of school. A total of 285 and 211 specimens were positive for pandemic (H1N1) 2009 virus and influenza A virus (H3N2), respectively. During April 2010, a second peak of pandemic (H1N1) 2009 was detected, and 303 cases were confirmed during epidemiologic weeks 13–16 (Figure A1).

By the time WHO declared the end of the pandemic on August 10, 2010, public health authorities recognized 1,805 cases of pandemic (H1N1) 2009 in Cuba. On the basis of laboratory results, during April 2009–2010, two peaks of pandemic (H1N1) 2009 were observed in Cuba.

During August 2010, Cuba experienced active transmission of seasonal influenza A (H3N2) virus, which displaced pandemic (H1N1) 2009 virus as the predominant virus. Similarly, seasonal influenza virus (H1N1) was displaced by pandemic (H1N1) 2009 virus. The last case of seasonal influenza (H1N1) detected in Cuba was in June 2009.

In this context, we believe that implementing national diagnostic algorithm enabled timely identification of the novel virus and effective monitoring of its circulation, even before international diagnostic protocols and reagents were available in Cuba. This study shows the need for nucleic acid amplification tools in laboratory diagnosis and surveillance of influenza viruses. As we prepare for future influenza pandemics, new and appropriate diagnostic methods and periodic assessment of influenza surveillance methods are needed as new information becomes available.

## Figures and Tables

**Figure A1:**
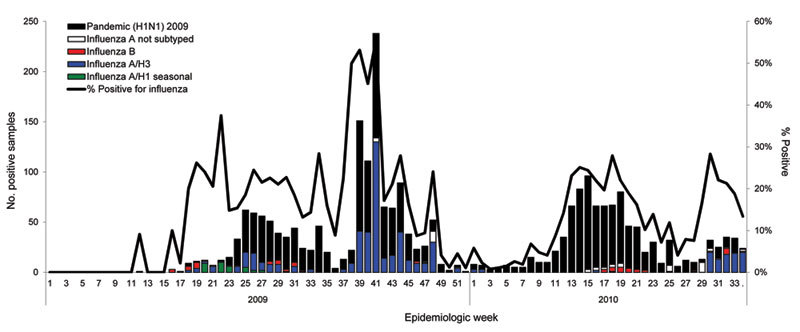
Influenza test results from weekly national summary, Havana, Cuba, epidemiologic week 1, 2009–epidemiologic week 34, 2010.

## References

[1] Generic protocol influenza surveillance, PAHO-CDC [cited 2011 Mar 4]. http://www.paho.org/English/AD/DPC/CD/flu-snl-gpis.pdf

[2] Panamerican Health Organization, World Health Organization. PAHO’s director newsletter. Issue 7. April 26, 2009 [cited 2011 Mar 3]. http://www.paho.org/English/D/D_DNewsLetters_eng.asp

[3] World Health Organization. WHO information for laboratory diagnosis of pandemic (H1N1) 2009 virus in humans [cited 2011 Mar 3]. http://www.who.int/csr/resources/publications/swineflu/diagnostic_recommendations/en/index.html

[4] Coiras MT, Perez-Brena P, Garcia ML, Casas I. Simultaneous detection of influenza A, B, and C viruses, respiratory syncytial virus, and adenoviruses in clinical samples by multiplex reverse transcription nested-PCR assay. J Med Virol. 2003;69:132–44. 10.1002/jmv.10255 12436489

[5] Coiras MT, Aguilar JC, Garcia ML, Casas I, Perez-Brena P. Simultaneous detection of fourteen respiratory viruses in clinical specimens by two multiplex reverse transcription nested-PCR assays. J Med Virol. 2004;72:484–95. 10.1002/jmv.20008 14748074 PMC7166637

[6] Pozo F, Garcia-Garcia ML, Calvo C, Cuesta I, Perez-Brena P, Casas I. High incidence of human bocavirus infection in children in Spain. J Clin Virol. 2007;40:224–8. 10.1016/j.jcv.2007.08.010 17904416 PMC7108365

[7] López-Huertas MR, Casas I, Acosta-Herrera B, Garcia ML, Coiras MT, Perez-Brena P. Two RT-PCR based assays to detect human metapneumovirus in nasopharyngeal aspirates. J Virol Methods. 2005;129:1–7. 10.1016/j.jviromet.2005.05.004 15961167 PMC7112860

[8] Valdés O, Piñón A, Acosta B, Savón C, González G, Arencibia A, Design and implementation of a molecular method for influenza A virus (H1N1) in Cuba. Rev Cubana Med Trop. 2011;63:15–20. 23437532

[9] Piñón A, Acosta B, Valdés O, Arencibia A, Savón C, González G, Cuban strategy for the molecular characterization of the pandemic influenza A virus (H1N1). Rev Cubana Med Trop. 2011;63:21–9. 23437533

[10] World Health Organization. CDC protocol of real-time RT-PCR for influenza A H1N1. April 28, 2009 [cited 2011 Mar 3]. http://www.who.int/csr/resources/publications/swineflu/CDCRealtimeRTPCR_SwineH1Assay-2009_20090430.pdf

